# Deep learning-based survival analysis of bladder cancer patients in the Putuo District, Shanghai, China

**DOI:** 10.3389/fonc.2025.1619309

**Published:** 2025-11-25

**Authors:** Wang Ruojing, Shen Yuan, Shi Feiya, Yang Lijuan, Qin Yicen

**Affiliations:** Department of Cancer Prevention and Systemic Regulation, Shanghai Putuo District Center for Disease Control and Prevention, Shanghai, China

**Keywords:** deep learning, bladder cancer, survival, logistic regression, 5-year

## Abstract

**Background:**

Bladder cancer poses significant health risks and necessitates effective public health management.

**Objective:**

To develop a deep-learning survival prediction model using TabNet and compare its performance with logistic regression.

**Methods:**

Data on bladder cancer patients were collected from the Putuo District subset of Shanghai Cancer Registration and Reporting System. A total of 620 patients were included, divided into a training cohort (n=434) and a validation cohort (n=186). Logistic regression analyses were conducted to identify risk factors, while the TabNet framework was used to develop a deep learning-based model. Model performance was evaluated using ROC curves, decision curve analysis, and calibration curves. Shapley Additive Explanations (SHAP) was applied to interpret feature importance.

**Results:**

Baseline characteristics showed no significant differences between the training and validation cohorts (P>0.05). The TabNet model demonstrated high discriminative ability in predicting both 5-year OS and CSS within the training cohort, with net benefits surpassing those of logistic regression, and showed good calibration. In the validation cohort, the TabNet model exhibited excellent performance in predicting 5-year OS and CSS. SHAP analysis revealed that age, T stage, and N stage were the most influential factors.

**Conclusion:**

The TabNet model showed robust performance in predicting bladder cancer survival, offering valuable insights for community-based management and follow-up strategies.

## Introduction

Bladder cancer ranks ninth in incidence and thirteenth in mortality among all malignancies. According to the Global Cancer Observatory, an estimated 613,791 new cases and 220,349 deaths were reported in 2022 (1). As the global population ages, the incidence of bladder cancer is projected to increase, posing continuous challenges to public health systems (2). In China, the improvement of medical record systems is exemplified by comprehensive records in Shanghai. The Shanghai Cancer Registry, renowned for its extensive scale and high-quality data, provides reliable information for analyzing cancer patient survival, mortality, and follow-up (3, 4). Tumor registry data from Putuo District offers a representative sample, facilitating effective in-depth analyses of bladder cancer.

Bladder cancer is recognized as one of the most economically demanding malignancies due to its complex treatment requirements and high recurrence rates (5, 6). Clinically, bladder cancer is classified into two distinct types: non-muscle-invasive bladder cancer (NMIBC) and muscle-invasive bladder cancer (MIBC), each with different management approaches and outcomes. The treatment typically involves substantial medical resources, including surgery, chemotherapy, and radiotherapy, resulting in significant direct medical costs. NMIBC management generally encompasses transurethral resection and intravesical therapy, whereas MIBC necessitates more aggressive interventions such as radical cystectomy combined with neoadjuvant or adjuvant systemic therapies. In addition to these costs, the disease imposes considerable socioeconomic impacts through lost productivity, prolonged unemployment, and the necessity for postoperative care. Furthermore, the management of bladder cancer patients involves periodic surveillance and ongoing medical care, which consume extensive healthcare and public health resources (7, 8). Surveillance is essential for ensuring treatment efficacy and monitoring disease progression, forming the basis for evaluating and adjusting therapeutic plans. However, these procedures significantly increase the complexity and cost of care. Thus, effective oncological management strategies must balance alleviating the financial burden on patients and their families with maintaining high-quality treatment outcomes. This necessitates healthcare providers and policymakers to optimize resource allocation, refine disease management strategies, and enhance the efficiency of healthcare services.

Accurate survival predictions enable the development of personalized follow-up plans, the optimization of examination schedules, and the organization of rehabilitation activities for bladder cancer patients. Individuals with a favorable prognosis might benefit from extended follow-up intervals to minimize unnecessary medical interventions, whereas high-risk patients necessitate intensive monitoring to detect recurrences or metastases promptly, thus ensuring timely treatment. Existing models for predicting bladder cancer survival rates exhibit varying predictive values and influencing factors. Traditional linear models, such as logistic regression, encounter limitations when handling large datasets and often fail to capture complex variable interactions (9, 10). Machine learning techniques have emerged to overcome these challenges, employing advanced algorithms to analyze multidimensional data patterns, including nonlinear relationships, thereby enhancing predictive accuracy (11).

Deep learning, as a subset of machine learning, has substantially advanced predictive modeling. These models excel in processing data and offer remarkable flexibility by automatically extracting and utilizing complex features, thereby enhancing predictive accuracy and generalization (12). Among these, the TabNet model stands out as a novel approach for tabular data, combining the strengths of decision trees and neural networks. This integration provides robust interpretability and efficient feature utilization, making it a powerful tool in predictive modeling (13).

The TabNet model has been successfully applied to prognostic analysis of liver metastases in rectal cancer patients, demonstrating high accuracy and reliability (14). Its application in bladder cancer survival analysis, however, has not yet been explored. This study employs the TabNet model to predict 5-year overall survival (OS) and cancer-specific survival (CSS) for bladder cancer patients in Putuo District, and to identify key risk factors affecting survival rates. This research is expected to provide a more precise prognostic tool for bladder cancer, thereby supporting public health decisions, optimizing follow-up schedules, and informing treatment strategies.

## Materials and methods

### Data sources

**Figure 1:**
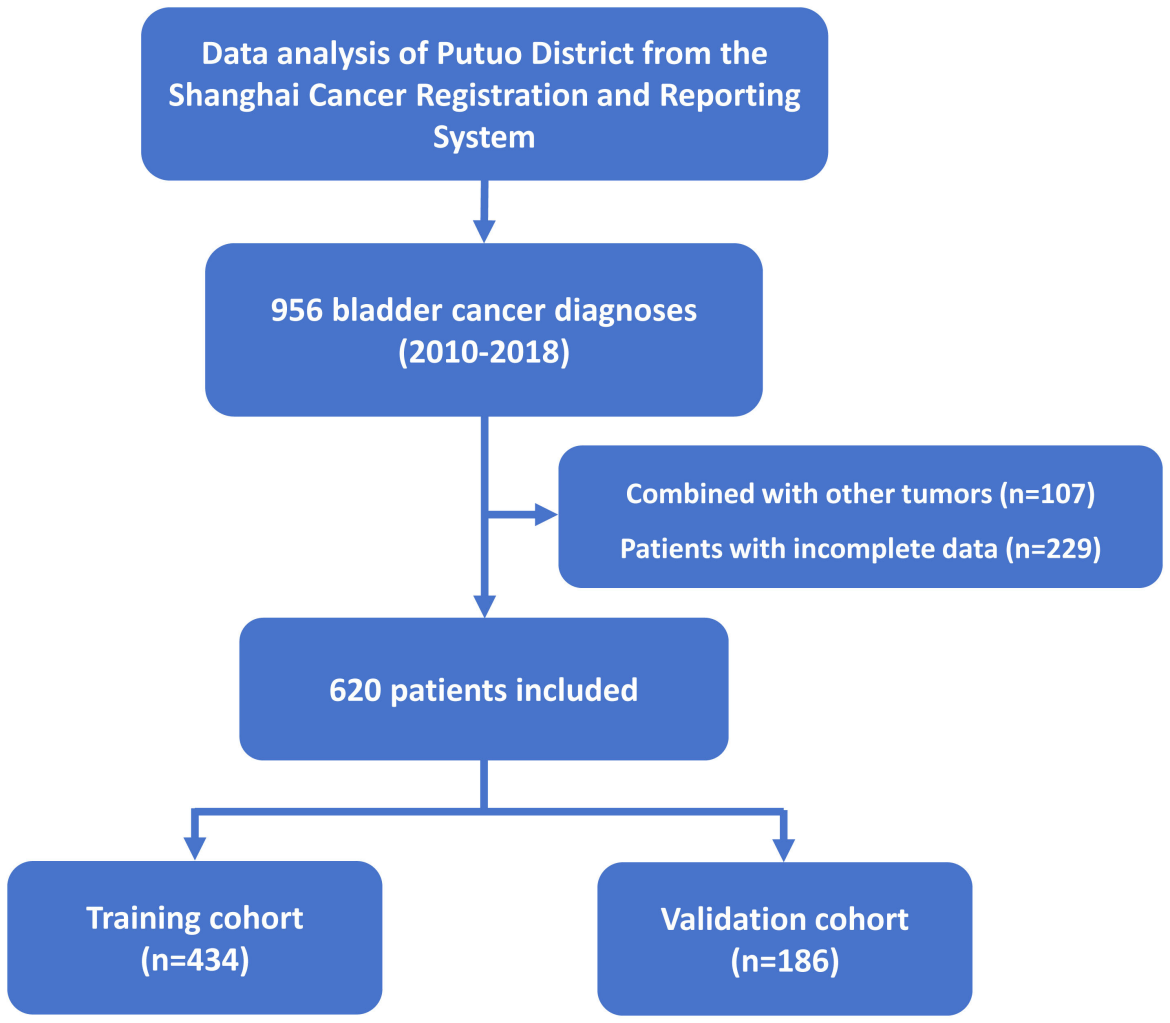
Flowchart of patient selection and cohort distribution.

Medical institutions in Shanghai have reported new malignancies within the household registered population since 2002, following the Shanghai Malignant Tumor Reporting Methods. These cases are systematically recorded in the Shanghai Cancer Registration and Reporting System and managed uniformly. Local primary healthcare institutions conduct consistent follow-ups based on the patients’ residential areas. Diagnoses are confirmed by secondary or higher-level medical institutions through histopathological and specialized diagnostic examinations. All malignancies in Shanghai are coded and classified according to the International Classification of Diseases (version 10, ICD-10), and the International Classification of Diseases for Oncology 3rd Edition (ICD-O-3). In this study, we statistically analyzed patients with ICD-O-3 codes C67.0-C67.9 in Putuo District between 2010 and 2018. Mortality data were obtained from the Putuo District all-cause death registration system, and population data were provided by the Putuo District Public Security Bureau. Data were reviewed, organized, and quality-assessed using IARCergTools software from the International Agency for Research on Cancer (IARC) and the International Association of Cancer Registries (IACR) (15, 16). This period was selected to ensure the availability of high-quality histopathological information and adequate follow-up time for survival analysis. Subsequently, patients with pathologically confirmed primary malignant bladder tumors and no other concurrent malignancies were included. Those with missing or incomplete data were excluded. Ultimately, 620 patients were included in the study, as illustrated in the flowchart (**Figure 1**). The dataset was randomly divided into training and validation cohorts in a 7:3 ratio. Ethical approval for this study was granted by the Ethical Review Committee of Shanghai Municipal Center for Disease Control and Prevention (2024-29). The requirement for informed consent was waived due to the retrospective design of the study and the use of anonymized and de-identified patient records.

### Key variables

The study analyzed 14 variables from the Putuo District data of the Shanghai Cancer Registration and Reporting System. These variables included gender (male, female), age (<60, 60–69, 70–79, >80), family history of bladder cancer (yes, no), smoking status (yes, no), tumor grade (low grade, high grade), histological type (urothelial carcinoma, non-urothelial carcinoma), AJCC stage (0a/0is/I, II, III, IV), T stage (Ta/Tis/T1, T2, T3, T4), N stage (N0, N+), M stage (M0, M1), surgery (yes, no), chemotherapy (yes, no), and radiotherapy (yes, no). The primary endpoint was 5-year OS, while the secondary endpoint was 5-year CSS.

### Model development

This study utilized the TabNet model to predict the outcomes of bladder cancer patients. Preprocessing entailed the normalization of all variables. Ordinal variables underwent ordinal encoding, nominal variables were subject to one-hot encoding, and binary variables were converted to 0 or 1. The TabNet model was implemented using the PyTorch TabNet framework. Hyperparameters were systematically optimized through an exhaustive grid search to minimize validation loss, with multiple parameter combinations evaluated. A five-fold cross-validation approach was used, dividing the training cohort into five parts, with each subset sequentially used as the validation cohort, thus ensuring model robustness and generalization. The model achieving the lowest validation loss across all folds was selected as the optimal model. Key hyperparameters are detailed in **Supplementary Table S1**.

### Statistical analysis

Data analysis was conducted using SPSS (IBM SPSS version 23.0, SPSS Inc). Univariate and multivariate logistic regression analyses were utilized to identify prognostic factors for survival in bladder cancer patients. The TabNet model was implemented using Python (Python version 3.8.6, Python Software Foundation). Performance evaluation included comparison of the area under the receiver operating characteristic (ROC) curve (AUC), accuracy, precision, recall, and F1 score between the TabNet and logistic regression models in the training cohort. Decision curve analysis and calibration curves were employed to evaluate clinical utility and predictive accuracy. Model performance was further assessed in the validation cohort to determine generalization and predictive accuracy on independent data. SHapley Additive exPlanations (SHAP) were applied to elucidate TabNet model predictions, quantify feature contributions, and enhance model transparency. Statistical significance was defined as p < 0.05.

## Results

### Baseline characteristics of the two groups

The cohort comprised 620 patients. The majority were male (77.42%), and most were aged 60 years and above. Regarding histological grade, 65.97% presented with low grade, while 34.03% presented with high grade. Urothelial carcinoma was the predominant histological type, accounting for 93.71% of cases, with non-urothelial carcinoma present in 6.29%. Bladder cancer was primarily diagnosed at an early stage, with only 6.45% of patients presenting with distant metastasis at diagnosis. Surgery was the primary treatment modality (82.42%), while radiotherapy was infrequently used (2.90%). The 5-year CSS rate was 81.80% in the training cohort and 82.26% in the validation cohort. Comprehensive demographic and clinical characteristics, along with comparative analysis, were detailed in **Supplementary Table S2**. No statistically significant differences were detected between the two cohorts across all variables (p > 0.05).

### Identification of prognostic factors with logistic regression

Univariate logistic regression analysis (**Supplementary Table S3**) identified age as a significant predictor, with 5-year OS and CSS risks increasing in the 60-69, 70-79, and >80 age groups (P<0.05). Patients with non-urothelial carcinoma had significantly lower 5-year OS and CSS compared to those with urothelial carcinoma (P<0.001). Higher tumor grade was associated with poorer OS and CSS (P<0.001). Tumor stages T2, T3, and T4 had significantly higher OS and CSS risks compared to Ta/Tis/T1 (P<0.05). Family history, smoking status, and N and M stages were also significant predictors (P<0.05). However, gender was not significant (P>0.05).

Further, multivariate logistic regression analysis (**Table 1**) demonstrated that 5-year OS and CSS in bladder cancer patients were significantly influenced by age, histology, tumor grade, T stage, and N stage (P<0.05). Survival rates were notably lower in patients over 80 years old, those with non-urothelial carcinoma, high tumor grade, and advanced T stage (P<0.05).

**Table 1:** Multivariate logistic regression analysis of 5-year OS and CSS.

Variable	OS			CSS		
	OR	95% CI	*P*	OR	95% CI	*P*
Family history	1.396	0.452-4.316	0.562	1.847	0.758-8.832	0.129
Smoking status	1.026	0.671-1.569	0.905	1.155	0.762-1.752	0.497
Gender	0.883	0.492-1.585	0.677	1.030	0.540-1.963	0.930
<60	Ref	/	<0.001	Ref	/	<0.001
60–69	2.795	1.210-6.458	0.016	7.051	1.942-25.606	0.003
70–79	8.091	3.705-17.668	<0.001	12.284	3.543-42.592	<0.001
>80	18.207	8.075-41.049	<0.001	22.431	6.396-78.660	<0.001
Histology	0.184	0.076-0.443	<0.001	0.203	0.086-0.478	<0.001
Grade	3.473	2.184-5.521	<0.001	2.729	1.657-4.493	<0.001
Ta/Tis/T1	Ref	/	0.001	Ref	/	0.016
T2	1.733	1.027-2.925	0.04	2.179	1.138-4.172	0.019
T3	2.181	1.075-4.425	0.031	2.969	1.314-6.708	0.009
T4	7.99	2.953-21.617	<0.001	4.044	1.488-10.985	0.006
N stage	2.721	1.264-5.861	0.011	2.587	1.169-5.726	0.019
M stage	1.133	0.433-2.960	0.799	1.349	0.580-3.140	0.487

OS, overall survival; CSS, cancer-specific survival; OR, odds ratio.

### Model training and cross-validation

**Figure 2:**
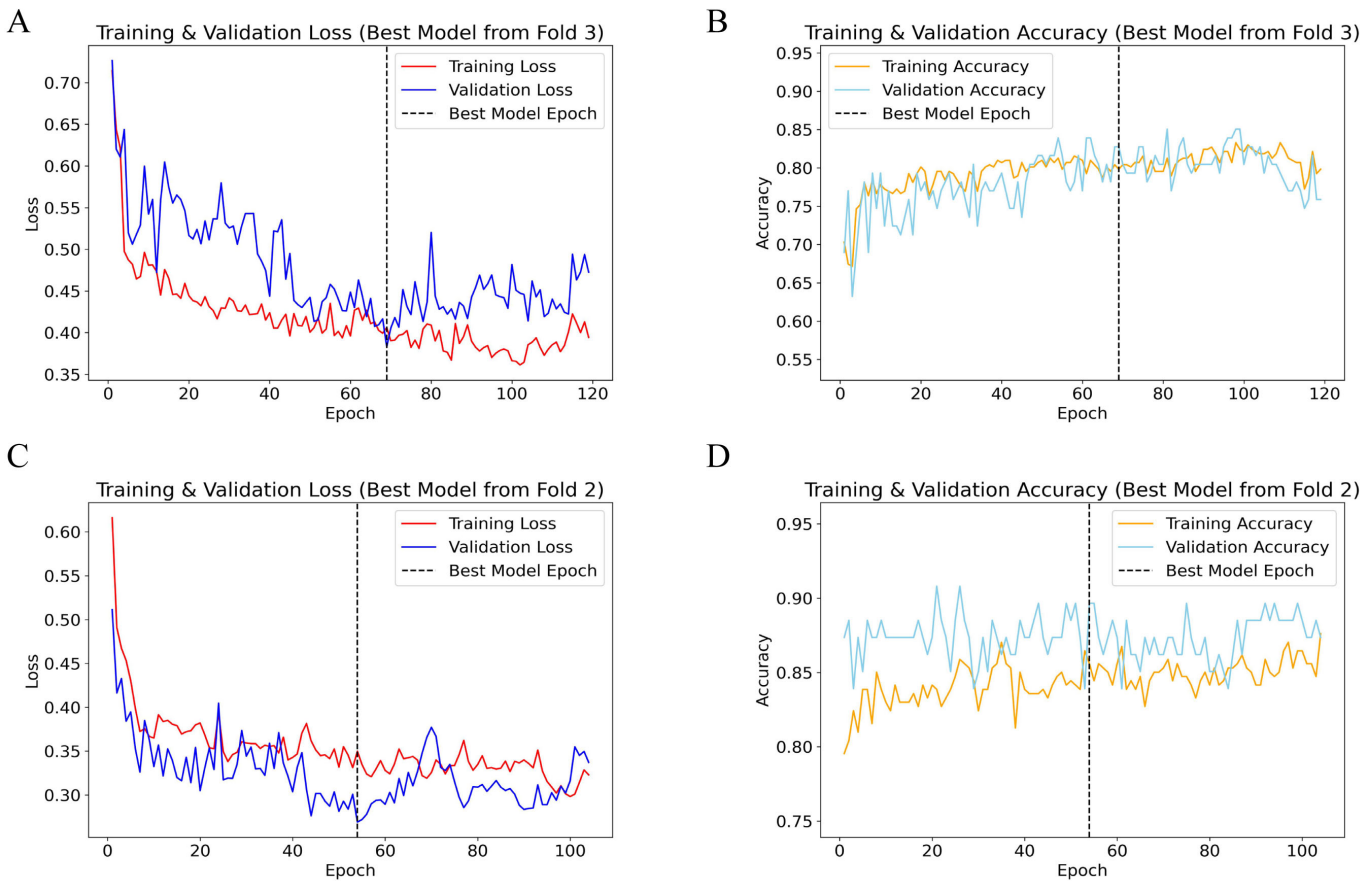
Performance plots of TabNet 5-fold cross-validation. **(A)** Training and validation loss for 5-year OS; **(B)** Training and validation accuracy for 5-year OS; **(C)** Training and validation loss for 5-year CSS; **(D)** Training and validation accuracy for 5-year CSS. OS, overall survival; CSS, cancer-specific survival.

During five-fold cross-validation, the optimal TabNet model for predicting 5-year OS was identified at the 65th epoch in the third fold (**Figures 2A, B**), achieving a validation loss of 0.387 and a validation accuracy of 0.842. Training loss decreased from 0.733 to 0.428, and training accuracy increased from 0.717 to 0.805. The consistent decrease in both training and validation losses, along with improved accuracies, indicates robust model performance and good generalization.

For predicting 5-year CSS, the best model was identified at the 53rd epoch in the second fold (**Figures 2C, D**), with a validation loss of 0.259 and a validation accuracy of 0.897. Both training and validation losses demonstrated a downward trend, accompanied by significant improvements in accuracy, reflecting high model accuracy and stability. The alignment of training and validation loss curves, without a notable increase in validation loss, indicates strong model generalization.

### Prediction of logistic regression and Tabnet in training cohort

**Figure 3:**
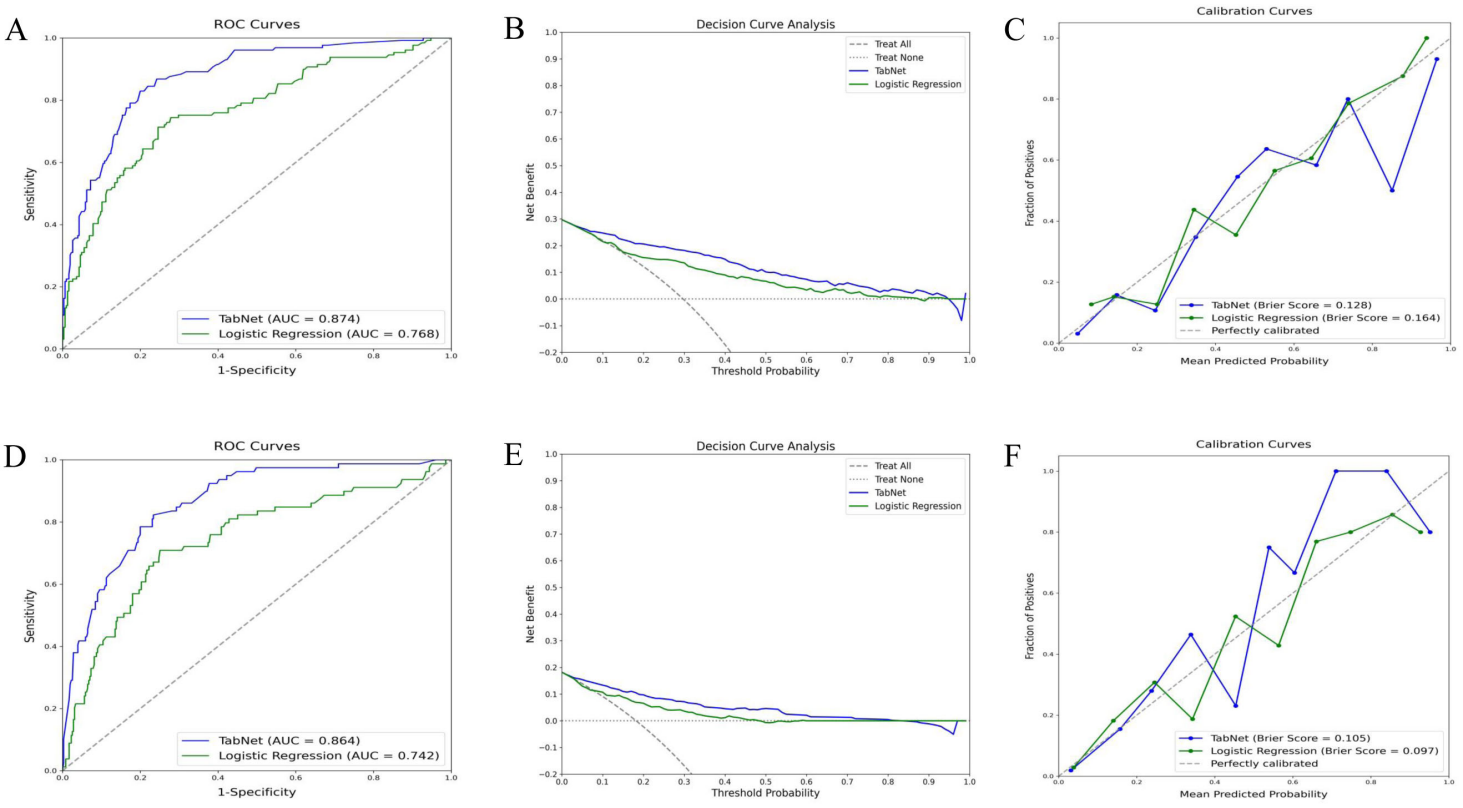
Predictive performance of TabNet and logistic regression in the training cohort. A-C: ROC curves **(A)**, decision curve analysis **(B)**, and calibration curves **(C)** for 5-year OS prediction. **(D-F)** ROC curves **(D)**, decision curve analysis **(E)**, and calibration curves **(F)** for 5-year CSS prediction. OS, overall survival; CSS, cancer-specific survival; ROC, receiver operating characteristic.

The TabNet model demonstrated superior discriminative ability in predicting 5-year OS, achieving an AUC of 0.874, compared to an AUC of 0.768 for the logistic regression model (**Figure 3A**). Decision curve analysis (**Figure 3B**) indicated that TabNet provided higher net benefits compared with logistic regression, across threshold probabilities ranging from 0 to 0.8. The calibration curve (**Figure 3C**) showed a Brier score of 0.128, indicating good calibration. TabNet outperformed logistic regression in accuracy, precision, recall, F1 score, and Kappa coefficient (**Table 2**).

**Table 2:** Performance comparison of predictive models in the training cohort.

Performance metrics	5-year OS		5-year CSS	
	TabNet	Logistic	TabNet	Logistic
AUC	0.874	0.768	0.864	0.742
Accuracy	0.804	0.770	0.864	0.811
Precision	0.750	0.641	0.750	0.333
Recall	0.512	0.512	0.380	0.038
F1 Score	0.608	0.569	0.504	0.068
Kappa	0.484	0.414	0.435	0.032
Brier Score	0.128	0.164	0.105	0.097

AUC, area under the curve. OS, overall survival; CSS, cancer-specific survival.

Similarly, for 5-year CSS prediction, the TabNet model exhibited an AUC of 0.864, surpassing an AUC of 0.742 for the logistic regression model (**Figure 3D**). Decision curve analysis (**Figure 3E**) demonstrated higher net benefits for TabNet at threshold probabilities ranging from 0 to 0.8. TabNet showed significantly better accuracy, precision, recall, F1 score, and Kappa coefficient. Despite a slightly better Brier score, the logistic regression model exhibited notably inferior overall predictive performance (**Figure 3F**).

### Prediction of TabNet in validation cohort

**Figure 4:**
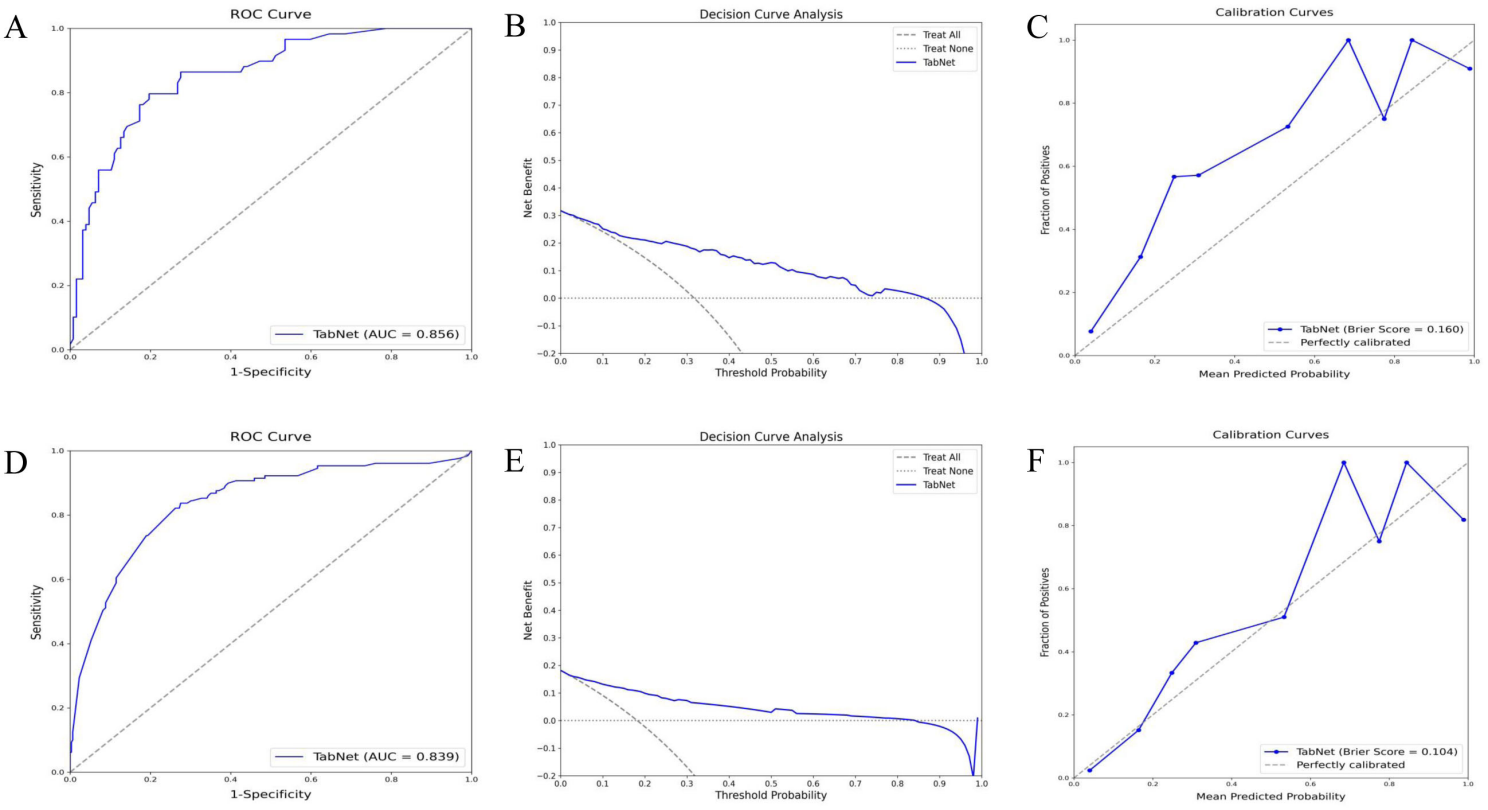
Validation performance of TabNet in predicting 5-year OS and CSS. A-C: ROC curves **(A)**, decision curve analysis **(B)**, and calibration curves **(C)** for 5-year OS prediction. **(D-F)** ROC curve **(D)**, decision curve analysis **(E)**, and calibration curve **(F)** for 5-year CSS prediction. OS, overall survival; CSS, cancer-specific survival; ROC, receiver operating characteristic.

The TabNet model demonstrated excellent performance in predicting 5-year OS and 5-year CSS within the validation cohort. ROC curve analysis (**Figure 4A**) for OS prediction showed an AUC of 0.856, indicating high discriminative ability. Decision curve analysis (**Figure 4B**) indicated significant net benefits for the TabNet model across various threshold probabilities. The calibration curve (**Figure 4C**) showed a Brier score of 0.160, reflecting good calibration. For 5-year CSS prediction, the TabNet model achieved an AUC of 0.839 (**Figure 4D**), indicating robust discriminative power. Decision curve analysis (**Figure 4E**) demonstrated substantial net benefits across threshold probabilities ranging from 0 to 0.7. The calibration curve (**Figure 4F**) revealed a Brier score of 0.104, indicating good calibration.

### SHAP analysis of TabNet model predictions

**Figure 5:**
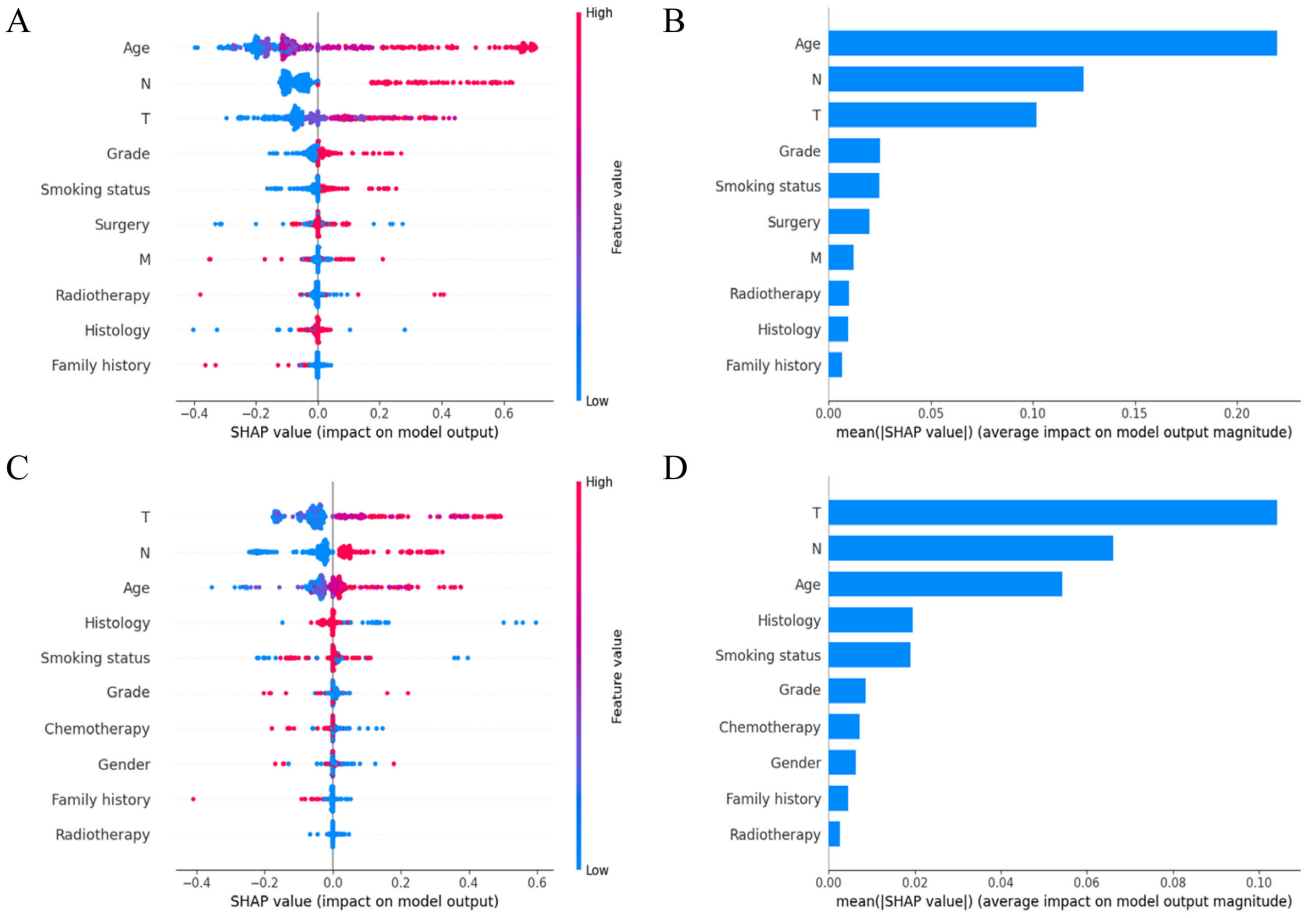
Variable importance in TabNet model. **(A)** SHAP summary plot of the top 10 features for predicting 5-year OS; **(B)** SHAP-based feature importance ranking for predicting 5-year OS; **(C)** SHAP summary plot of the top 10 features for predicting 5-year CSS; **(D)** SHAP-based feature importance ranking for predicting 5-year CSS. SHAP, shapley additive explanations; OS, overall survival; CSS, cancer-specific survival.

SHAP analysis elucidated the significance and specific impacts of various features in the TabNet model. As shown in **Figure 5A**, higher feature values (red) and lower feature values (blue) were observed to have different impacts on the model’s output. Positive SHAP values indicated an increased predicted probability of death, while negative SHAP values indicated an increased predicted probability of survival. Age significantly affected the model’s predictions, with increased age correlating with a reduced probability of survival. The N stage and T stage were also critical, with higher stages indicating more advanced disease and poorer prognosis. Additionally, tumor grade and smoking status also had a notable impact. The average SHAP value plot (**Figure 5B**) revealed that age, N stage, and T stage had the greatest impact on 5-year OS predictions, while tumor grade and smoking status exhibited a moderate effect.

For predicting 5-year CSS, the SHAP summary plot (**Figure 5C**) illustrated the impact of each feature on the model’s output, with red indicating higher feature values and blue indicating lower feature values. The T stage, N stage, and age emerged as key determinants of the model’s predictions, with higher T and N stages and older age associated with worse survival outcomes. The average SHAP value plot (**Figure 5D**) identified T stage, N stage, and age as having the most notable average effects on 5-year CSS predictions, with histology and smoking status having a moderate influence.

## Discussion

Bladder cancer, the second most common malignancy in the genitourinary system after prostate cancer, has rising incidence and mortality rates, imposing significant burdens on society and healthcare systems (17). Effective predictive models are crucial for community-based cancer management, patient follow-up, and healthcare resource allocation. This study utilized data from the Putuo District within the Shanghai Cancer Registration and Reporting System to develop and validate an individualized survival prediction model for bladder cancer patients using deep learning algorithms. The TabNet model demonstrated enhanced performance in predicting 5-year OS and CSS compared to logistic regression, achieving higher accuracy and better calibration. In the validation set, TabNet demonstrated superior performance in ROC curves, decision curve analysis, and calibration curves, indicating high clinical utility. SHAP analysis identified age, N stage, and T stage as the primary factors affecting 5-year OS and CSS. These findings provide a basis for improving community-based cancer surveillance and facilitating more efficient population health management for bladder cancer patients.

The 5-year survival rates for bladder cancer patients exhibit regional variations globally. Europe reports an age-standardized relative 5-year OS of approximately 70% (18). In the United States, the average 5-year OS is 77%, with 19% of patients succumbing to bladder cancer (19). Data from China show an increase in the 5-year survival rate from 67.3% to 71.6% between 2003 and 2015, comparable to international levels (20). This study found a 5-year OS rate of 70.48% and a CSS rate of 81.94%, slightly lower but generally in line with the national data. According to the National Central Cancer Registry (NCCR) of China (21), 91.4% of bladder cancers are urothelial carcinoma, with approximately 55-60% classified as low-grade tumors. These proportions align with our findings, indicating the representativeness and reliability of the sample. The cancer registry provides high-quality, comprehensive data, enabling effective monitoring of cancer incidence and survival rates, thereby supporting the development and evaluation of cancer prevention and control strategies.

Traditionally, clinicians have relied on the American Joint Committee on Cancer (AJCC) staging guidelines, using T, N, and M stages for preliminary prognosis assessment (22). However, this system fails to account for demographic factors or treatment modalities, which significantly influence outcome prediction. Consequently, researchers have developed more comprehensive predictive models. Zhang et al. (23). integrated TNM staging with clinical parameters to construct a nomogram for visualizing survival rates, achieving a C-index of 0.813 for 5-year OS, thereby demonstrating superior predictive performance compared to the AJCC-TNM staging. Similarly, He et al. (24). developed a nomogram based on the Surveillance, Epidemiology, and End Results (SEER) database to predict CSS for postoperative bladder cancer at the population level, yielding a C-index of 0.823. Despite the acceptable predictive accuracy of these models, certain limitations exist. The complexity of the calculations and the challenges in addressing collinearity among variables may impact the stability of the results. In this study, logistic regression analysis was used to predict 5-year OS, resulting in an AUC of 0.768, and 5-year CSS, resulting in an AUC of 0.742. These findings are consistent with previous research, indicating a moderate level of predictive capability and highlighting the potential for further improvement.

The continuous advancement of artificial intelligence has led to the development of innovative predictive tools. Leveraging multi-omics data from large-scale databases (GEO, TCGA, and IMvigor210), ensemble machine learning approaches have successfully constructed robust risk stratification models, demonstrating significant value in predicting treatment response and immunotherapy outcomes for bladder cancer patients (25). In a study involving 161,227 bladder cancer patients, Bhambhvani et al. (26). employed clinical-pathological data and sociodemographic variables to train an artificial neural network (ANN) for predicting 5-year CSS, achieving an AUC of 0.81, which was more accurate than traditional multivariate models. Although ANNs are proficient at capturing complex patterns and nonlinear relationships, they present challenges in transparency, feature selection, and susceptibility to overfitting. In comparison, deep learning utilizes more intricate neural network structures to enhance the extraction and processing of complex features. These advanced models excel in handling high-dimensional and large-scale data, effectively mitigating overfitting risks (27, 28). In this study, the TabNet deep learning model demonstrated AUCs of 0.874 for 5-year OS and 0.864 for CSS, significantly exceeding the performance of logistic regression models. The moderate F1 and Kappa values reflect the sensitivity of these metrics to class imbalance (80% survival vs. 20% death), while the high AUC and positive decision curve analysis confirm strong discrimination ability for risk stratification. These findings underscore the potential of TabNet in prognostic prediction for bladder cancer and provide strong support for its application in community-based cancer management and surveillance. Indeed, the attention mechanism in TabNet facilitates automatic feature selection, addresses collinearity issues, and enhances model transparency and interpretability, making it particularly suitable for population health management.

Bladder cancer is a complex disease influenced by various factors, significantly impacting survival rates. Age at diagnosis is a well-known predictor of poor OS and CSS in bladder cancer (29, 30). A study indicates that cancer mortality is 15 times higher in individuals aged ≥65 compared to those under 65 (31). In our study, age was identified as the most significant factor affecting 5-year OS. This can be attributed to the decline in immune function in elderly patients, reducing their resistance to tumors. The presence of chronic diseases further increases the risk of treatment complications. Additionally, older patients are often diagnosed at more advanced stages, delaying optimal treatment (32). In another study based on data from the SEER database, Wang et al. (33). found that age, T stage, and N stage were the important prognostic factors, which is consistent with our findings. The T stage indicates the extent of tumor invasion, with higher stages correlating with deeper bladder wall invasion and poorer prognosis (34). The N stage reflects lymph node involvement, with higher stages suggesting greater metastatic risk, complicating treatment, and lowering survival rates (35).

Moreover, our study identified smoking and tumor grade as additional prognostic factors with a considerable degree of influence. Numerous studies have demonstrated a strong association between smoking and the development of bladder cancer, identifying it as a significant risk factor (36–38). Approximately 30-50% of bladder cancer deaths are attributed to smoking across various populations (39). Although smoking status ranked as a moderate predictor in our SHAP analysis, its modifiable nature distinguishes it from other prognostic factors. This suggests that smoking cessation interventions integrated into survivorship care could potentially improve outcomes, representing a practical target for both clinical and community-level prevention strategies. Similar to 5-year OS, 5-year CSS in our study was primarily influenced by age, T stage, and N stage, with smoking and histological features also impacting patient prognosis. Patients with non-urothelial carcinomas, such as adenocarcinoma and squamous cell carcinoma, have poorer prognoses, likely due to the aggressive nature of these histological types and their poorer response to standard treatments (40).

Prognostic modeling in bladder cancer has achieved significant advances, with Li et al. (41) developing a validated nomogram for predicting overall survival in postoperative high-grade bladder urothelial carcinoma patients using SEER data and external validation cohorts, while Bhambhvani et al. demonstrated that ANN could achieve enhanced discriminative performance in 5-year survival prediction (26). In the present study, we employed TabNet architecture integrated with SHAP analysis to enhance model interpretability. The SHAP framework enables decomposition of model predictions into additive feature attributions, revealing not only which variables influence prognosis but also the magnitude and directionality of their effects across different patient profiles. This transparency facilitates personalized risk assessment by allowing clinicians to understand the specific factors driving individual patient predictions, thereby supporting evidence-based treatment planning and informed patient counseling. An ideal prognostic tool should incorporate readily available variables with significant predictive value while maintaining interpretability, and our TabNet model with SHAP analysis achieved satisfactory predictive performance alongside transparent feature attribution.

This study has several limitations. First, the lack of data on surgical methods, patient comorbidities, and socioeconomic status may affect prognostic assessment. Efforts were made to mitigate this limitation by incorporating a comprehensive set of other relevant variables to enhance the robustness of the analysis. Second, excluding patients with incomplete data may introduce selection bias. However, this approach was necessary to maintain data integrity and analytical accuracy, thereby preventing potential errors due to missing data. Third, this model was developed and validated exclusively using data from Putuo District. Although the Shanghai Cancer Registry ensures high-quality population-based surveillance with comprehensive follow-up, the geographic confinement to a single district raises concerns about generalizability. Regional variations in bladder cancer epidemiology, including risk factor distributions and clinical presentations, may influence model performance across different populations. External validation using datasets from geographically and demographically diverse populations is essential to assess the model’s transferability and clinical utility. Prospective validation efforts would help determine the model’s broader applicability and inform necessary adaptations for specific regional contexts. Fourth, our cohort of 620 patients is relatively small for deep learning. However, rigorous validation demonstrated satisfactory generalization, and TabNet’s superior performance over logistic regression justified this approach. Larger multi-center datasets would further strengthen model robustness and enable broader generalizability assessment. Fifth, our comparison was limited to TabNet and logistic regression. Comprehensive benchmarking with additional machine learning methods (XGBoost, Random Forest, multilayer perceptron) would provide valuable insights and represents an important direction for future research with larger multi-center datasets.

## Conclusion

The deep learning-based TabNet model shows great potential in predicting survival outcomes for bladder cancer patients, offering advantages such as handling high-dimensional data and providing robust performance. This model can be a valuable tool for community healthcare workers to identify high-risk patients who require more intensive follow-up and resource allocation. Age, T stage, and N stage emerged as the most significant prognostic factors, highlighting their critical role in determining patient outcomes and providing guidance for developing targeted community intervention strategies. External validation across diverse populations is warranted to establish the model’s generalizability before broader clinical implementation.

## Data Availability

The original contributions presented in the study are included in the article/**Supplementary Material**. Further inquiries can be directed to the corresponding author/s.

## Supplementary material

Supplementary Table 1 TabNet model hyperparameters and training protocol.

Supplementary Table 2 Demographic and clinical data for training and validation cohorts.

Supplementary Table 3 Univariate logistic regression analysis of 5-year OS and CSS.
