# Supplementary material for: Deep learning-based survival analysis of bladder cancer patients in the Putuo District, Shanghai, China
Source: Front Oncol. 2025 Nov 25;15:1619309. doi: 10.3389/fonc.2025.1619309 (PMC12685632; doi:10.3389/fonc.2025.1619309)
Supplement: Supplementary Table 1 — TabNet model hyperparameters and training protocol. [file Table1.docx]

**Supplementary materials**

Table S1. TabNet model hyperparameters and training protocol

| Category | Parameter | Value |
| --- | --- | --- |
| Model architecture | Decision steps | 8 |
|  | Features per decision step | 8 |
|  | Step size | 0.2 |
|  | Independent sparsity coefficient | 1×10^-5^ |
| Optimization | Loss function | Binary cross-entropy |
|  | Optimizer | Adagrad |
|  | Learning rate | 0.1 |
| Training protocol | Batch size | 16 |
|  | Virtual batch size | 8 |
|  | Cross-validation folds | 5 |
|  | Early stopping patience | 50 epochs |
|  | Maximum training epochs | 1000 per fold |
| Model selection | Selection criterion | Lowest validation loss across all folds |

Table S2. Demographic and clinical data for training and validation cohorts

| Characteristic |  | Total cohort (n=620) | Training cohort (n=434) | Validation cohort (n=186) | P |
| --- | --- | --- | --- | --- | --- |
| Gender, n% | Male | 480 (77.42) | 337 (77.65) | 143 (76.88) | 0.834 |
|  | Female | 140 (22.58) | 97 (22.35) | 43 (23.12) |  |
| Age, n (%) | <60 | 155 (25.00) | 110 (25.35) | 45 (24.19) | 0.704 |
|  | 60–69 | 154 (24.84) | 107 (24.65) | 47 (25.27) |  |
|  | 70–79 | 173 (27.90) | 123 (28.34) | 50 (26.88) |  |
|  | >80 | 138 (22.26) | 94 (21.66) | 44 (23.66) |  |
| Family history, n (%) | Yes | 21 (3.39) | 14 (3.23) | 7 (3.76) | 0.735 |
|  | No | 599 (96.61) | 420 (96.77) | 179 (94.62) |  |
| Smoking status, n (%) | Yes | 235 (37.90) | 163 (37.56) | 72 (38.71) | 0.786 |
|  | No | 385 (62.10) | 271 (62.44) | 114 (61.29) |  |
| Grade, n (%) | Low grade | 409 (65.97) | 282 (64.98) | 127 (68.28) | 0.553 |
|  | High grade | 211 (34.03) | 152 (35.02) | 59 (31.72) |  |
| Histology, n (%) | Urothelial carcinoma | 581 (93.71) | 406 (93.55) | 175 (94.09) | 0.801 |
|  | Non-urothelial carcinoma | 39 (6.29) | 28 (6.45) | 11 (5.91) |  |
| Stage, n (%) | 0a/0is/I | 421 (67.90) | 295 (67.97) | 126 (67.74) | 0.965 |
|  | II | 74 (11.94) | 56 (12.90) | 18 (9.68) |  |
|  | III | 72 (11.61) | 46 (10.60) | 26 (13.98) |  |
|  | IV | 53 (8.55) | 37 (8.53) | 16 (8.60) |  |
| T stage, n (%) | Ta/Tis/T1 | 440 (70.97) | 310 (71.43) | 130 (69.89) | 0.411 |
|  | T2 | 95 (15.32) | 68 (15.67) | 27 (14.52) |  |
|  | T3 | 51 (8.23) | 32 (7.37) | 19 (10.22) |  |
|  | T4 | 34 (5.48) | 24 (5.53) | 10 (5.38) |  |
| N stage, n (%) | N0 | 510 (82.26) | 361 (83.18) | 149 (80.11) | 0.405 |
|  | N1-3 | 110 (17.74) | 73 (16.82) | 37 (19.89) |  |
| M stage, n (%) | M0 | 580 (93.55) | 405 (93.32) | 175 (94.09) | 0.553 |
|  | M1 | 40 (6.45) | 29 (6.68) | 11 (5.91) |  |
| Surgery, n (%) | Yes | 511 (82.42) | 359 (82.72) | 152 (81.72) | 0.629 |
|  | No | 109 (17.58) | 75 (17.28) | 34 (18.28) |  |
| Chemotherapy, n (%) | Yes | 269 (43.39) | 191 (44.01) | 78 (4194) | 0.899 |
|  | No | 351 (56.61) | 243 (55.99) | 108 (58.06) |  |
| Radiotherapy, n (%) | Yes | 18 (2.90) | 12 (2.76) | 6 (3.22) | 0.745 |
|  | No | 602 (97.10) | 422 (97.24) | 180 (96.77) |  |
| 5-year CSS, n (%) |  | 508 (81.94) | 355 (81.80) | 153 (82.26) | 0.891 |
| 5-year OS, n (%) |  | 437 (70.48) | 305 (70.28) | 132 (70.97) | 0.863 |

OS, overall survival; CSS, cancer-specific survival; OR, odds ratio.

Table S3. Univariate logistic regression analysis of 5-year OS and CSS

| Variable | OS | | |  | CSS | | |
| --- | --- | --- | --- | --- | --- | --- | --- |
|  | OR | 95% CI | P |  | OR | 95% CI | P |
| Family history | 1.526 | 1.237-1.882 | 0.042 |  | 2.325 | 1.771-3.053 | 0.018 |
| Smoking status | 2.337 | 1.815-3.099 | 0.015 |  | 3.155 | 2.252-4.420 | 0.007 |
| Gender | 0.989 | 0.604-1.621 | 0.966 |  | 1.100 | 0.67-1.807 | 0.706 |
| <60 | Ref | / | <0.001 |  | Ref | / | <0.001 |
| 60–69 | 2.909 | 1.392-6.081 | 0.005 |  | 7.131 | 2.065-24.63 | 0.002 |
| 70–79 | 8.897 | 4.489-17.634 | <0.001 |  | 16.244 | 4.920-53.631 | <0.001 |
| >80 | 17.529 | 8.707-35.289 | <0.001 |  | 27.895 | 8.446-92.126 | <0.001 |
| Histology | 0.176 | 0.077-0.401 | <0.001 |  | 0.228 | 0.117-0.444 | <0.001 |
| Grade | 2.154 | 1.417-3.276 | <0.001 |  | 2.329 | 1.540-3.522 | <0.001 |
| Ta/Tis/T1 | Ref | / | <0.001 |  | Ref | / | <0.001 |
| T2 | 1.709 | 1.063-2.749 | 0.027 |  | 2.455 | 1.347-4.473 | 0.003 |
| T3 | 4.067 | 2.473-6.688 | <0.001 |  | 5.134 | 2.805-9.399 | <0.001 |
| T4 | 23.929 | 11.914-48.063 | <0.001 |  | 12.824 | 6.730-24.4262 | <0.001 |
| N stage | 9.149 | 5.351-15.644 | <0.001 |  | 7.430 | 4.729-11.672 | <0.001 |
| M stage | 3.405 | 1.856-6.246 | <0.001 |  | 3.953 | 2.309-6.768 | <0.001 |

OS, overall survival; CSS, cancer-specific survival; OR, odds ratio.
